# Supplementary material for: Responses of Soil Microbial and Nematode Communities to Various Cover Crop Patterns in a Tea Garden of China
Source: Int J Environ Res Public Health. 2022 Feb 25;19(5):2695. doi: 10.3390/ijerph19052695 (PMC8910492; doi:10.3390/ijerph19052695)
Supplement: Supplementary file 1 [file ijerph-19-02695-s001.zip › ijerph-1602634-supplementary.pdf]

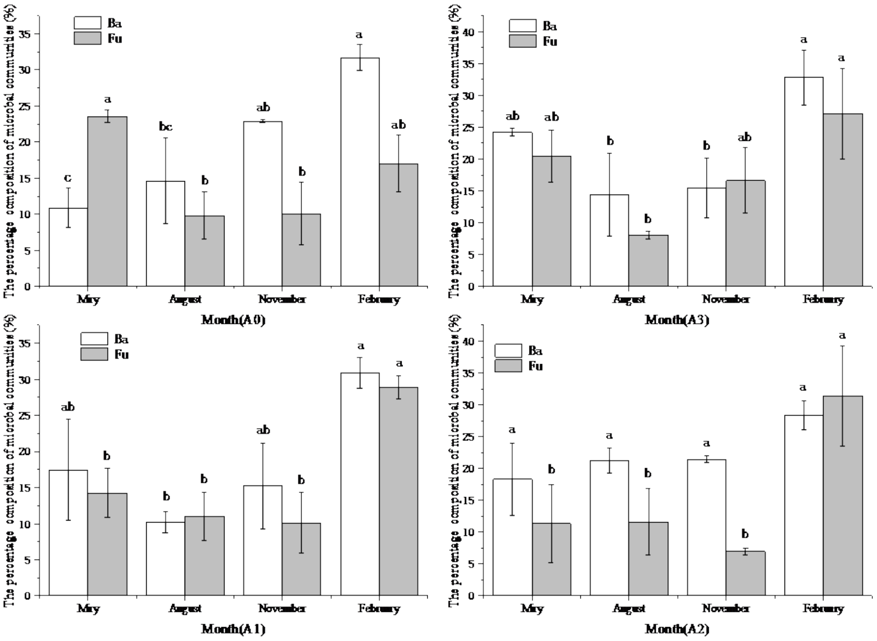

Figure S1 The percentage of soil fungi and common bacteria in different months under various cover crop patterns.

Note: A0 represents naturally covered of bare land; A1 represents mixed planting with two-species; A2 represents four-species, andA3 represents eight-species. The same below. Different lowercase letters indicate significant differences between different months (*p*<0.05).

Table S1 The sowing weight of covered crops in a tea plantation

Cover crop	A1	A2	A3
Lolium perenne L.	15	7.5	6.25
Trifolium repens L.	10	5	3.3
Poa annua L.		7.5	6.25
Vicia villosa Roth.		5	3.3
Trifolium pretense L.			3.3
Festuca rubra L.			0.8
Cosmos bipinnata Cav.			0.8
Zinnia elegans Jacq.			0.8

Note: The unit of the weight in the covered crops is g/m^2. A0 represents naturally covered of bare land; A1 represents mixed planting with two-species; A2 represents four-species, andA3 represents eight-species. The same below.

Table S2. Biological characterization of PLFAs

Microbial community species	Phospholipid fatty acids
Gram-positive bacteria (G+)	i15:0;a15:0;i16:0;i17:0;a17:0;i18:0;a18:0;18:1w11c
Gram-negative bacteria (G-)	16:1w9c;16:1w11;17:1w9;17:1w10c;i17:1w11c;18:1w8t;18:1w9t;18:1w11;18:1w13c;cy16:0;cy17:0;cy19:0
Bacteria (B)	10:0;13:0;14:0;15:0;9Me15:0;16:0;17:0;17:1w8;18:0;18:1w8;20:0;20:4w5,8,11,14;20:4w5,8,11,14c;20:5w5,8,11,14,17
Fungi (F)	18:1w7;18:1w9;18:1w9c;18:2w8,11;18:2w9,1;18:2w10,13t
Actinomycetes (A)	2Me17:0;10Me17:0;14Me18:0;10Me19:0;11Me19:0

Table S3. Two-way ANOVA analyses of cover crop patterns and time effects on soil physical

and chemical properties.

Edaphic physicochemical factors	Source	d.f.	Sum Sq	F	*p*
Water content of soil(WCS) (%)	Month	3	166.866	149.794	< 0.001
	Cover crop patterns	3	0.588	0.528	0.666
	M*C[a]	9	0.558	0.500	0.863
pH	Month	3	0.093	7.491	0.001
	Cover crop patterns	3	0.234	18.875	< 0.001
	M*C	9	0.010	0.788	0.629
Total nitrogen(TN) (g/kg)	Month	3	0.140	13.307	< 0.001
	Cover crop patterns	3	0.025	2.382	0.088
	M*C	9	0.007	0.685	0.716
Total phosphorus(TP) (g/kg)	Month	3	0.022	1.567	0.217
	Cover crop patterns	3	0.178	12.955	< 0.001
	M*C	9	0.004	0.321	0.962
Nitrate nitrogen(NO_3^-) (mg/kg)	Month	3	14.610	15.765	< 0.001
	Cover crop patterns	3	1.669	1.801	0.167
	M*C	9	0.969	1.045	0.428
Ammonium nitrogen(NH_4^+) (mg/kg)	Month	3	30.236	9.705	< 0.001
	Cover crop patterns	3	5.249	1.685	0.190
	M*C	9	2.430	0.780	0.636
Available phosphorus(AP) (mg/kg)	Month	3	255.937	7.227	0.001
	Cover crop patterns	3	20.354	0.575	0.636
	M*C	9	30.569	0.863	0.566
Organic matter(OM) (g/kg)	Month	3	0.784	32.795	< 0.001
	Cover crop patterns	3	0.072	3.003	0.045
	M*C	9	0.022	0.939	0.506

a. M*C: Month* Cover crop patterns.

Table S4. Two-way ANOVA of cover crop patterns and time effects on soil microbial

communities.

Microbial community	Source	d.f.	Sum Sq	F	*p*
Total PLFA	Month	3	502.467	8.673	< 0.001
	Cover crop patterns	3	90.824	1.568	0.216
	M*C[a]	9	52.769	0.911	0.528
G+	Month	3	49.986	15.933	< 0.001
	Cover crop patterns	3	0.150	0.048	0.986
	M*C	9	2.690	0.857	0.571
G-	Month	3	55.298	4.016	0.016
	Cover crop patterns	3	13.548	0.984	0.413
	M*C	9	5.007	0.364	0.944
B	Month	3	74.762	10.966	< 0.001
	Cover crop patterns	3	15.191	2.228	0.104
	M*C	9	5.397	0.792	0.626
F	Month	3	561.533	199.831	< 0.001
	Cover crop patterns	3	59.569	21.199	< 0.001
	M*C	9	4.151	1.477	0.239
A	Month	3	2.939	9.518	< 0.001
	Cover crop patterns	3	0.434	1.406	0.259
	M*C	9	0.572	1.851	0.097

a. M*C: Month* Cover crop patterns. G+: gram-positive bacteria; G-: gram-negative bacteria; B: bacteria; F: fungi; A: actinomycetes.

Table S5 Two-factor ANOVA of cover crop modes and months on soil nematode communities

Nematode community	Source	df	Sum Sq	F	P
Totol number of nematodes	Month	3	399659.8	196.823	<0.001
	Cover crop patterns	3	244658.3	120.488	<0.001
	M*C[a]	9	17373.38	8.556	<0.001
Ba	Month	3	136141.942	64.276	<0.001
	Cover crop patterns	3	158292.432	74.734	<0.001
	M*C	9	16069.140	7.587	<0.001
Fu	Month	3	46485.460	72.317	<0.001
	Cover crop patterns	3	7904.026	12.296	<0.001
	M*C	9	559.350	0.870	0.561
Op	Month	3	5610.596	32.315	<0.001
	Cover crop patterns	3	788.818	4.543	<0.001
	M*C	9	329.683	1.899	0.088
Pp	Month	3	1874.487	8.978	<0.001
	Cover crop patterns	3	314.710	1.507	0.231
	M*C	9	582.140	2.788	0.016

Note: Ba - bacterivores; Fu - fungivores; Op -omnivores / predators; Pp - plant parasites.
